# Social networks and the conservation of fish

**DOI:** 10.1038/s42003-022-03138-w

**Published:** 2022-02-28

**Authors:** David Villegas-Ríos, David M. P. Jacoby, Johann Mourier

**Affiliations:** 1 Instituto Mediterráneo de Estudios Avanzados (CSIC-UiB), Departamento de Ecología y Recursos Marinos, C/Miquel Marqués 21, 07190 Esporles, Islas Baleares Spain; 2 Instituto de Investigaciones Marinas (CSIC), Departamento de Ecología y Recursos Marinos, Eduardo Cabello 6, 36208 Vigo, Pontevedra Spain; 3 Lancaster Environment Centre, Lancaster University, Lancaster, LA1 4YQ UK; 4 UMS 3514 Plateforme Marine Stella Mare, Université de Corse Pasquale Paoli, 20620 Biguglia, France

**Keywords:** Conservation biology, Behavioural ecology

## Abstract

Despite our critical dependence on aquatic wildlife, we lack a complete understanding of the drivers of population stability and structure for most fish species. Social network analysis has been increasingly used to investigate animal societies as it explicitly links individual decision-making to population-level processes and demography. While the study of social structure is of great ecological interest, it is also potentially important for species of economic value or of conservation concern. To date however, there has been little focus on how social processes are likely to influence the conservation of fish populations. Here we identify applications for how a social network approach can help address broad fish conservation themes such as population structure, biological invasions or fisheries management. We discuss the burgeoning opportunities offered and challenges still faced by current technologies to integrate social network approaches within fish conservation.

## Abstract (web summary)

The study of animal societies has been increasingly explored with social network analysis. In this Perspective, the importance of these networks in directing conservation efforts of fish is highlighted.

## Sociality in fish

Most fish species depend on social cues to make important life-history decisions such as finding a mate, initiating migration, evading predation, acquiring resources, or optimizing foraging strategies^1^. Our current understanding of fish societies reflects decades of work on a relatively small number of model species. In freshwater systems, studies on a number of Gasterosteiform, Cyprinodontiformes, and Cichliform fishes have revealed stable partner associations that can result in highly structured societies^2, 3^. In the marine realm, significant insight has been gained through the early study of coral reef fishes^4^. Since then, the emergence of cooperative behaviors and social reproductive strategies, in a number of reef teleost fishes, has attracted significant research attention (e.g., ref. ^5^). Further, considerable focus has been invested exploring how collective behavior of fish leads to highly coordinated schools^6^.

**Fig. 1:**
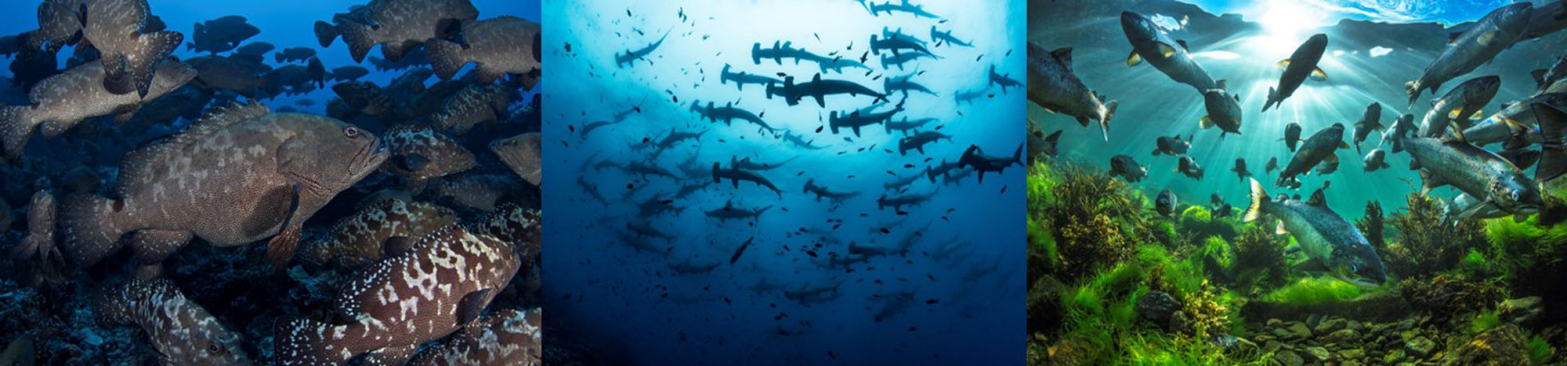
The social lives of fish. Examples of fish species of conservation concern that may benefit from the use of social network analysis such as (from left to right) spawning marbled groupers (*Epinephelus polyphekadion*), schooling scalloped hammerhead sharks (*Sphyrna lewini*), or Atlantic salmon (*Salmo salar*). Photo credits: marbled grouper by T. Vignaud, scalloped hammerhead sharks by S.J. Pierce and Atlantic salmon by A. Rikardsen. All photos published with permissions from authors.

Interestingly, many aspects of the life-histories of species of economic relevance or conservation concern also have a strong social component (Fig. 1). These, however, are typically poorly understood, despite the potential benefits of this information for their management and conservation^7^. For example, species such as cod, plaice, or groupers rely on social learning for the transmission and maintenance of migration routes^8^, and reproductive behavior has a social component in multiple species of elasmobranch and teleost fishes^9,10^.

To date, the vast majority of studies of sociality in fish have focused at the level of the group and over short time scales^9^ preventing an understanding of the structure and stability of social interactions within groups. Here we discuss the opportunities offered by social network analysis (SNA) to better integrate the sociality of fish into conservation and fisheries science, identify tangible research needs and discuss present and future opportunities offered by developing technologies to achieve this goal.

## Social networks of fish

Exploration of social network structure requires considerable data on the repeated interactions or associations of many individuals. For long-lived, air-breathing marine mammals, such as cetaceans, this has been successfully achieved through the direct observation of animal interactions recorded at the surface using years of photo identification data (e.g., ref. ^11^). The social network structure of fishes has classically been explored under controlled laboratory, or semi-wild conditions using model species^9,12^. These studies have demonstrated patterns of social structuring including social preferences, phenotypic and behavioral assortment, and community structure^12^.

**Fig. 2:**
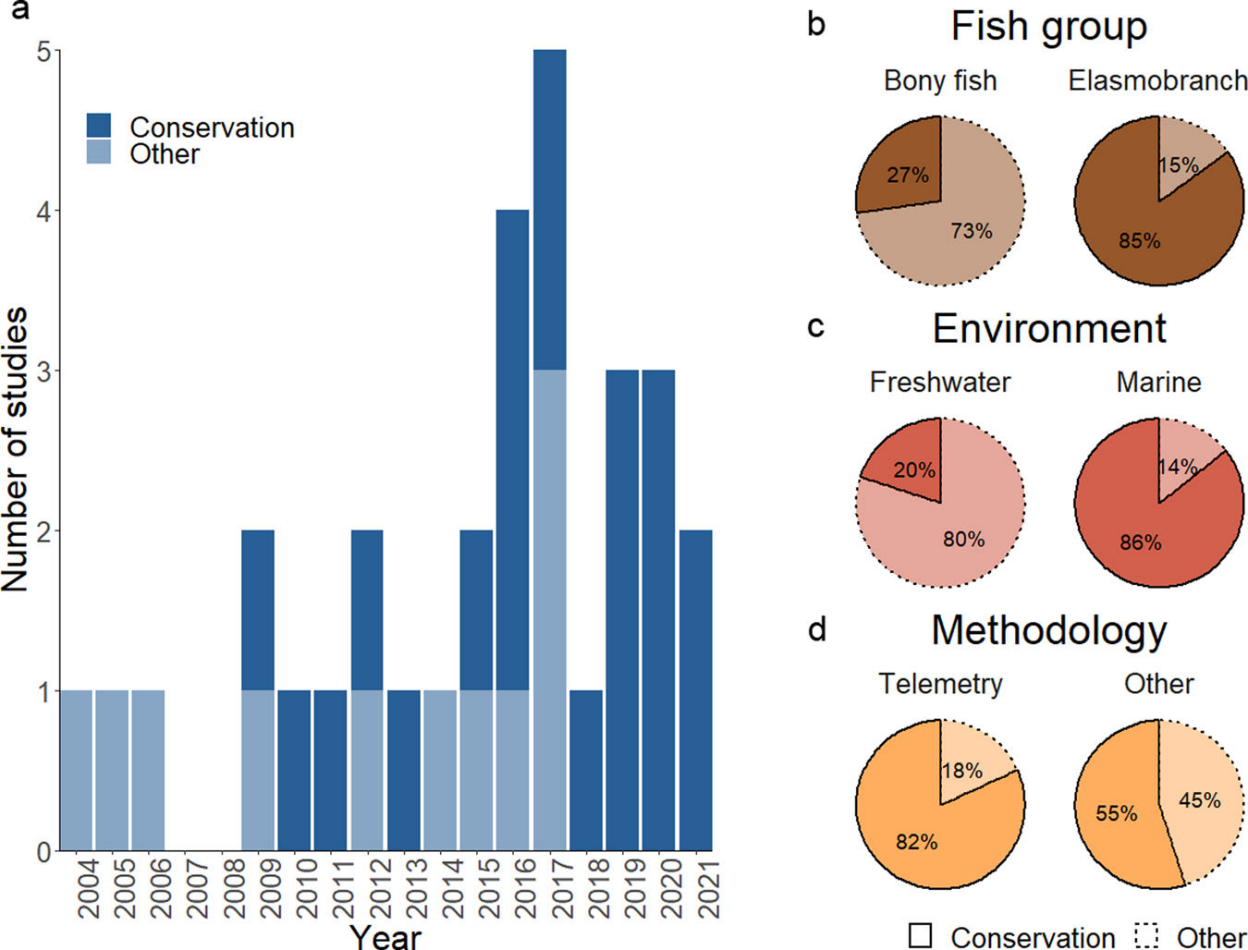
Wild studies of fish social networks. **a** Several studies have investigated the social networks of fish in the wild in the last two decades of which ~68% focused on species of conservation or commercial interest (“Conservation”). The remainder focused on model species or species of least conservation concern (“Other”). The percentage of studies related to species of conservation concern varied between **b** fish groups, **c** environments, and **d** methodology used. Data for this figure were obtained from an extensive search in SCOPUS and by consulting the reference list of the most relevant reviews on the topic.

Monitoring populations in their natural environment, however, is important to understand the emergence and structure of social networks within the context of other effects that might influence behavior such as environmental variation^6,13,14^. To date, there remains just a few studies that have attempted to do this (Fig. 2; summarized in Supplementary Table 1). Early studies used direct observations or capture-recapture methods to infer social associations within freshwater wild habitats over short periods of time. Over the last decade, however, there has been a tendency towards using telemetry techniques (Supplementary Table 1) to infer social associations in the wild and over time periods more relevant to understanding the broader ecological and evolutionary patterns, which are important for conservation (i.e., several months to years; Supplementary Table 1). Yet, the number of studies using telemetry techniques is relatively few and restricted predominantly to studies on elasmobranchs (Fig. 2; Supplementary Table 1). Moreover, the precision of recording direct associations with some telemetry methods such as passive acoustic tracking is still rather low (but see ref. ^15^ for a new high-resolution tracking application). Consequently, our ability to integrate knowledge of social behavior into the sustainable management or conservation of target fish populations, remains limited.

## Tracking social structure of wild fish populations

**Fig. 3:**
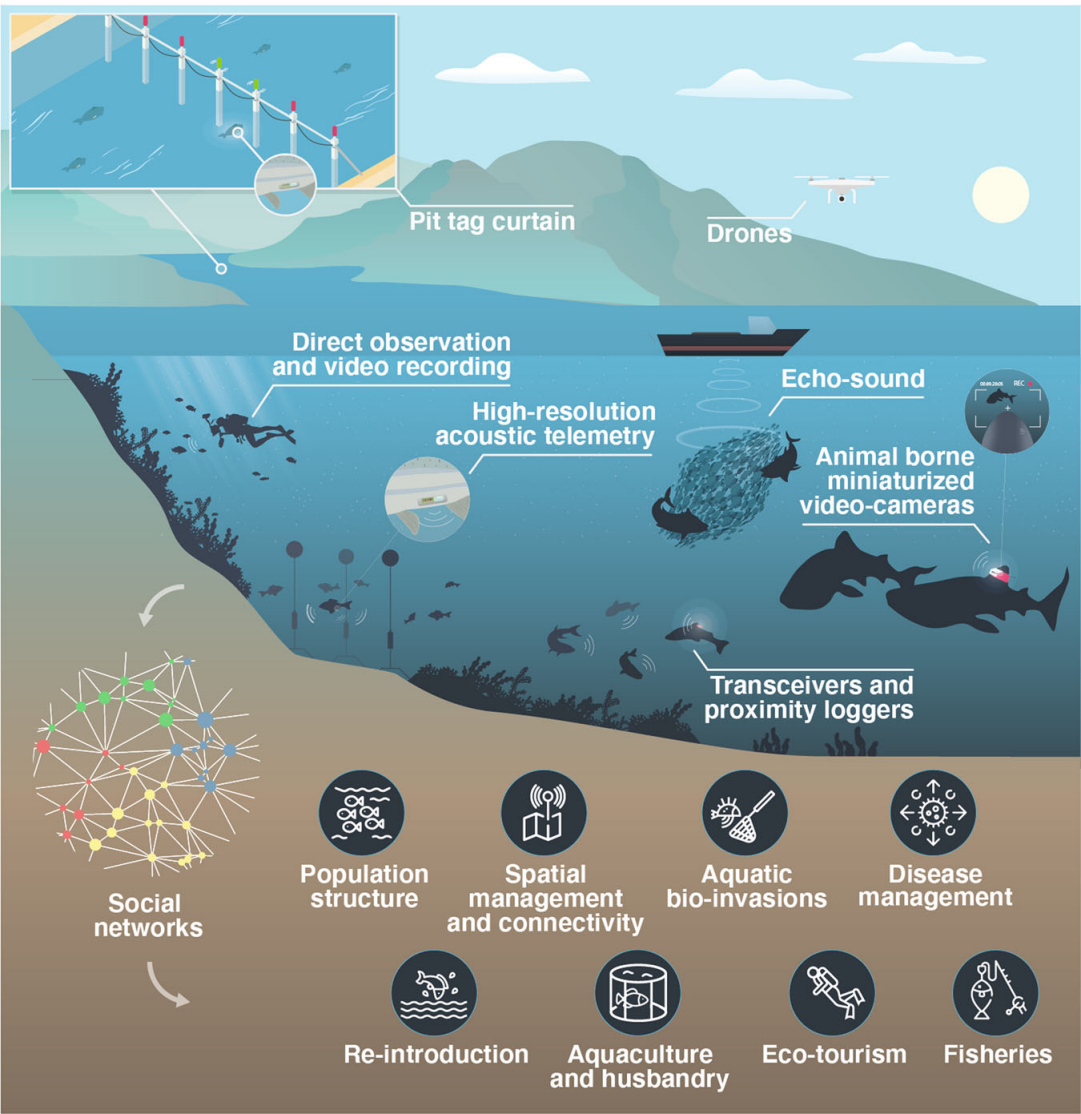
Available technologies to track social interactions in wild aquatic ecosystems. There are a number of technologies currently available to investigate the social structure of fish depending on the habitat (e.g., marine vs. freshwater, coastal vs. oceanic, shallow vs. deep waters). The social networks constructed based on those technologies can help us understand many aspects of fish conservation. All technologies have their advantages and disadvantages but importantly, all only capture a proportion of the underlying population-level social network.

Until recently, tracking the behavior of numerous fish simultaneously in their natural habitat, and in high resolution, has been logistically challenging and/or prohibitively expensive. However, this information is crucial to building robust social networks^16^. Today, new technologies and analytical tools offer several ways of recording the associations of fish in the wild (Fig. 3).

Collective migration into rivers for example, can be tracked with passive integrated transponder (PIT) tags, where individuals migrating together are detected as they pass through PIT ‘curtains’. While PIT tag systems do not monitor fish positions continuously, they are well-suited to recording movements through specific corridors, gates, or bottlenecks. In coastal and shallow waters, some fish species can be directly observed or recorded using videography or photo ID methods. Computer-vision techniques are now sufficiently advanced to be able to reproduce individual tracks from videos, as well as identify associations^17^.

Acoustic telemetry now offers a number of opportunities^18^. Mobile peer-to-peer technologies, such as animal-borne acoustic telemetry transceivers and proximity loggers offer considerable promise, as they can be used to detect associations as animals range over larger spatial scales^19^. Currently, however, these devices remain rather large, expensive, power hungry, and reliant on the retrieval of the logger for data recovery, tending to result in typically short-term deployments on a small number of large animals. One alternative for site-attached species is high-resolution spatial positioning using acoustic tracking, which can track many individuals remotely and simultaneously with high accuracy (<1 m) as long as they move within the boundaries of a receiver array^20^. So far high-resolution tracking has been limited to freshwater ecosystems (but see ref. ^21^), and there is a need to test this equipment in different marine environments where the transmission of high-frequency acoustic signals is more challenging.

Attempts to study fish social networks with telemetry often rely on the assumption that individuals in close proximity are associating with one another^22^. Consequently, there is a need to go beyond inferring associations in favor of actually measuring them, as well as the nature of any interactions between individuals. This can be achieved by the use of sensor transmitters that measure the behavioral response associated with the spatial associations. Also, aquatic megafauna can carry customized tracking devices with video cameras that record the nature of interactions (in the short-term) with other conspecifics in the open ocean^23^. However, further improvements are needed to overcome, for instance, the significant size and weight restrictions on the use of combined telemetry and video camera, animal-borne packages. Other burgeoning techniques such as echo-sounders or aerial and underwater drones are used to investigate collective behavior or identify fish aggregations^24^, a prerequisite for sociality to emerge^6^.

With these developments and ongoing challenges in mind we now address first why and then how the integration of SNA might significantly improve our ability to conserve and manage wild fish populations.

## Benefits of integrating social network structure within fish conservation

Although the explicit integration of social behavior in conservation and management is still relatively uncommon, there are some examples from the terrestrial literature showing the effective integration of SNA in species conservation. Perhaps the area where SNA has been most widely applied in terrestrial systems is disease ecology and transmission. For instance, contact networks were used to explore transmission potential of devil facial tumor disease in the endangered Tasmanian devil, *Sarcophilus harrisii*^25^. Combined with spatial information, SNA has also proven useful to track parasite transmission and load in the sleepy lizard, *Tiliqua rugosa*^26^. SNA has also helped to identify individuals of great apes that, given their social status, are more likely to facilitate the spread of pernicious diseases (e.g., ref. ^27^). Finally, SNA has been widely used to investigate patterns of infection in dairy cattle showing how variations in management regimes (e.g., grazing access, milking routine) substantially alter cattle contact patterns and potentially infection transmission^28^.

In aquatic systems, studies assessing the role of fish social behavior on conservation have focused on processes at the individual or dyadic level. A benefit of the SNA approach is that it measures social processes at the individual, group, and population level across different spatial and temporal scales.

Here we propose that a social network approach might facilitate a better understanding of how fish social behavior affects eight important aquatic conservation themes (Table 1). SNA has the potential to address important questions relating to population structure. Fish behavior, such as aggregations, agonistic behavior, fission–fusion dynamics, or trait-based dispersal, can bias our estimates of effective population size due to biased sampling^29,30^. For instance, in sticklebacks, less social individuals were shown to enter a sampling trap that already contained conspecifics sooner than social counterparts^31^. Importantly, many wildlife conservation problems are directly linked to demography^32,33^. Demography in turn is influenced by social behavior. Thus, understanding population-level social structure quantified through metrics that measure connectivity, community structure and stability, for example, is crucial for addressing whether human perturbations (e.g., fishing, pollution, noise) affect disproportionally one aspect of the population more than others (e.g., size, age class, or sex)^34^. To date, SNA has been already used to reveal meta-population structure, characterize group dynamics and identify communities in species such as sand tiger sharks^29^, blacktip reef sharks^35^, and manta rays^36^.

**Table 1:** Benefits of social network analysis for fish conservation.

Conservation theme	Social mechanisms that can be addressed with social networks	Conservation outcome
Population structure	• Aggregation behavior. • Assortative group formation (e.g., sexual segregation). • Fission–fusion behavior. • Trait-based dispersal.	• Avoid or acknowledge biased population estimates due to biased sampling. • Detect human perturbations affecting disproportionally one segment of the population. • Clearer definition of effective population size.
Spatial management and connectivity	• Spatially determined social interactions.	• More effective design of aquatic protected areas. • Identification of key habitats or spots where animals socialize (e.g., cleaning stations, mating grounds, feeding aggregations). • More accurate estimates of effective population (social + spatial) connectivity. • Identifying the habitat or environmental conditions that promote sociality.
Aquatic bio-invasions	• Integration of invasive species into native social structures. • Competition between native and invasive species. • Identify traits that favor social integration.	• Optimize strategies to manage fish bio-invasions (e.g., targeted eradications).
Fish disease management	• Identify hubs of infection. • Identify individuals that act as vectors or super spreaders of diseases or parasites.	• Monitor spread of diseases on both wild and semi-wild settings. • Develop and optimize strategies to manage fish diseases (e.g., targeted eradications, targeted vaccination).
Re-introductions	• Integration of reintroduced individuals into natural social structures. • Competition between reintroduced individuals and natural populations. • Identify traits that favor social integration.	• Detect why stocked individuals may not succeed in integrating with natural populations. • Improving housing and transport conditions.
Aquaculture and husbandry	• Alteration of behavior due to captivity conditions. • Competition for food.	• Improve welfare conditions. • Increase productivity of farmed animals. • Improved feeding efficiency.
Eco-tourism	• Tourism-driven alteration of fish social structure (aggregation or repulsion).	• Responsive eco-tourism management. • Alteration of provisioning regime/methods.
Fisheries	• Aggregation behavior. • Correlations between social position and fitness. • Changes in social structure due to fishing activities. • Changes in social structure due to fishing regulatory measures (MPAs, size limits).	• Integration of sociality into stock assessment models. • Predict fisheries-induced selection on social behavior. • Understand the capacity to restore social structure through different management actions.

Conservation themes that might benefit from adopting a social network approach, indicating some of the potential mechanisms through which social behavior may affect each conservation theme, and the conservation outcomes.

Social interactions do not take place randomly in space, and the existence of spatially determined social interactions may have important implications for spatial management and connectivity. In combination with spatial information, SNA may help in detecting areas where animals preferentially socialize (e.g., for mating or feeding purposes) revealing which habitats or environmental conditions favor sociality^37^. This information can be then used by managers to implement or adapt existing spatial protection measures such as marine protected areas (MPAs) in order to include social hotspots and to more effectively protect ecosystem functioning^36,38^. Information on social and spatial connectivity may also be used to make more accurate estimates of population connectivity^39^ and therefore delineate management units or stocks.

A critical step in the study of aquatic bio-invasions is the assessment of how invasive species integrate or compete with local communities. Being able to integrate with the social circle of native communities, together with the capacity to adapt to the new environment, is key for the success of an invasion^40^. Using SNA Beyer et al.^40^ showed that invasive sunbleak *Leucaspius delineatus* display stronger social connections with native species than the native species show with each other. This type of study may also reveal the potential traits of successful invaders that can be used by managers for instance to perform targeted eradications. Importantly, invasive species may not only bring new parasites but also spread them faster among the native communities raising additional conservation concerns. In fact, another promising application of SNA is in fish disease management, i.e., studying the diffusion of parasites, pathogens and diseases in both wild and stocked species^41^. SNA can be used to disentangle social and non-social influences on fish disease propagation and to identify hubs of infection or individuals that could act as super spreaders. In the aquatic realm, this approach could be particularly useful in aquaculture settings, where potential super spreaders can be individually identified and removed or vaccinated. In the wild, tracking technologies combined with SNA can be used to investigate the capacity of migratory individuals to act as super spreaders, as migration can increase contact rates, exposure, susceptibility, and competence^42^, or how altered spatial behavior will affect disease transmission in natural populations^43^.

Successful reintroduction (including re-stocked and translocated individuals) depends in part upon individual ability to move through structurally complex environments, avoid predators, forage efficiently but also interact socially^30^. Here, SNA can help to explore if and how reintroduced individuals integrate into, or compete with local communities, which will ultimately determine the success of those conservation actions^44^. Such an approach may also prove useful for inferring whether introduced fish display the same level of sociality observed in wild conspecifics, possibly triggering corrective actions such as enriching of rearing environments to better facilitate post-release integration.

Tracking social interactions in captive or semi-wild conditions can also benefit aquaculture and husbandry by generating information about rearing conditions that optimize animal welfare and productivity. For instance, social hierarchies and competition can be investigated with SNA to understand how feeding regime influences competition and thus optimize feeding efficiency^45^. Acoustic telemetry has already been used to assess the effect of enriched environments on the behavior of gilthead seabream *Sparus aurata* kept in cages^46^ and this approach could easily be extended to understand the effect of farming conditions (e.g., stocking density) on social structure. Another application of SNA to fish farming would be the investigation of efficiency of social learning techniques commonly used for instance, to introduce pelleted food^47^.

Eco-tourism practices such as food provisioning can alter the behavior of aquatic animals by promoting the aggregation of animals that otherwise would not associate. For instance, a recent study reported the altered social structure of tiger sharks *Galeocerdo cuvier* at provisioning sites in The Bahamas, characterized by more frequent and more random aggregations at dive sites compared to non-human-impacted locations^48^. Elsewhere, the presence of humans (e.g., scuba divers) can repel normally local animals away from tourist sites^36^. Both processes (attraction and repulsion) can eventually alter important life-history aspects of fish such as visits to spawning or feeding grounds, mating behavior or predator–prey relationships. SNA can be used to investigate changes in all these aspects simultaneously. In practice, this might lead to more responsible eco-tourism management (e.g., reduce noise impacts or curb scuba-diving at specific locations or times), and/or alter existing provisioning methods.

**Fig. 4:**
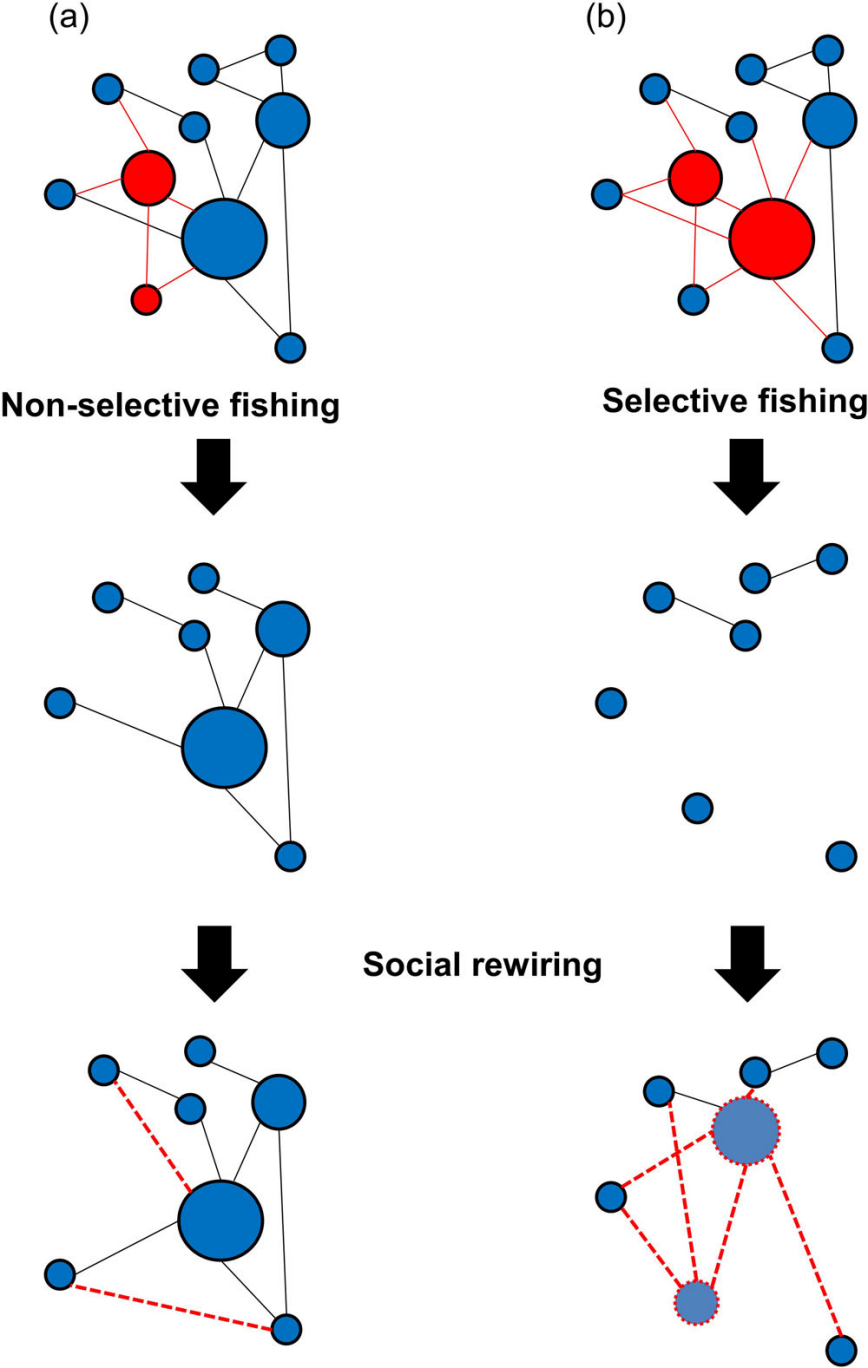
Fishing effects on fish social structure. Through correlations between life-history, behavior, physiology, and social traits, fishing can range from **a** non-selective to **b** highly selective on social network position, with direct effects on the topology of the resulting network and the demography of the populations. Social rewiring dynamics after fishing will determine if and when the social network can reach an equilibrium state. Red nodes and solid red edges represent individuals, and their associations, removed by the fishery. Red dashed edges represent newly created associations after the fishing event. Blue nodes represent non-harvested individuals.

Fisheries science has typically not considered the links between social behavior, population density and population dynamics. Fishing may disrupt social structure through direct effects, i.e., direct demographic change^49^. Direct effects may occur through the non-selective removal of individuals and the social interactions they were involved in (Fig. 4a). However, the effects of fishing on network structure may be exacerbated if fishing is selective on social behavior, i.e., if the fished individuals are especially highly connected, dominant or perform important social functions^49^ (Fig. 4b). This could result from direct selection on social behavior (i.e., when the social phenotype affects survival to the fishery), or through indirect selection on correlated traits (e.g., selection on larger or bolder individuals that in turn are more socially connected). Evidence of direct fishing-induced selection on social position is scarce. Recently, Guerra et al.^50^, showed how industrial fishing is selective against individual behaviors that produce large groups, likely eroding social structure of the affected populations. Fishing is also predicted to affect network structure through indirect effects by changing the connectivity of the remaining (i.e. non harvested) individuals, leading to either positive or negative effects on the population. This social rewiring can be particularly relevant when fishing is selective on social position and the remaining individuals compete for the social role of some of the harvested individuals^49^. This kind of network dynamics certainly occur naturally in wild populations (e.g., in hermaphrodite fish species where sex change is socially mediated^51^). However, the rate at which demographic changes due to fishing occur, and the resulting changes in social structure, are typically much faster and can therefore have a greater impact on network stability and population resilience, than the dynamic changes in social interaction often measured by social network studies^49,52^.

## Social networks and fish conservation in practice

Given the potential of SNA to impact the conservation of fish, we here outline what we believe to be four key areas where tangible advances can be made to bridge the gap between social behavior and fish conservation.

### Characterizing social structure

Recording social interactions using existing technology can provide readily useful information for a variety of conservation themes. At present, there are a myriad of ways in which social interactions can be recorded or inferred depending on the behavior and mobility of the fish species (see section Tracking social structure of wild fish populations). Based on interactions information, networks can be constructed to understand population structure. Community detection algorithms (i.e., algorithms used to evaluate how groups of nodes are clustered) might then usefully be applied to detect group composition and measure the number of communities across age-classes and in response to changing environmental conditions^53^. Network metrics, such as assortativity (that measure the tendency of individuals with the same attributes to interact with each other) or network modularity (that measures the strength of division of a given network into modules), can be used to understand the degree of mixing or segregation among social groups. Alternatively, data on social interactions, combined with information about pathogens and transmission rates, can also feed into network-based diffusion analyses to better understand how parasites or diseases may diffuse in the target populations. Combined with movement, habitat and/or environmental information, which is typically available in fish spatial ecology studies, researchers can start to identify areas of special interest for fish conservation that also consider social ecology. For example, multilayer network approaches may be used to analyze interlayer edges, spatial and social centrality and other indicators of combined social and spatial connectivity^38,54^. Other methods such as INLA multi-matrix animal models can also be used in combination with data on social interactions, demography, and the environment to identify key habitats where fish socialize (e.g., cleaning stations, feeding grounds)^55^, helping to measure how sociality may affect persistence via its effects on survival or reproduction^32^.

### Understanding the link between social network position and fitness

Links between social network position (e.g., centrality, connectedness), fish attributes (e.g., body size, sex, genotype), and fitness-related traits (e.g., survival, reproductive success) remain virtually unexplored in wild fish populations. However, these links are critical to understand feedback loops between social position and demography, and can determine population resilience and dynamics^49^. Such important links have been revealed in marine mammals, primates and birds. For instance, more socially integrated male killer whales, *Orcinus orca*, had a significantly lower risk of mortality than peripheral males^56^; female rhesus macaques *Macaca mulatta* that maintained strong connections to favored partners had the highest relative survival probability^57^ and females of brown-headed cowbirds, *Molothrus ater* with stronger familiarity preferences in autumn laid more eggs in the spring^58^. In practice, aquatic biologists could adopt a whole-field approach and use current telemetry techniques to track social interactions and survival at the same time in the wild^59,60^, and relate it to individual traits. To accomplish this, the appropriate study systems need careful selection. We suggest that a reasonable starting point would be to work with existing infrastructure that tracks species of limited movement (e.g., reef fish;^21^) or that move in enclosed (e.g., lakes^61^) or semi-enclosed areas (e.g., fjords;^53^) where social interactions can be tracked continuously for long periods. Much of such infrastructure is already in place with a spatial resolution that is sufficient to infer social interactions. Since tracking all the individuals of the population is virtually impossible for most species, researchers should be careful in selecting the network metrics that enable valid inference of social position and structure using partial networks (i.e., networks constructed based on a subset of the population)^16^. Establishing the aforementioned links can, for instance, be useful for tracking the impact of bio-invasions, by identifying the traits associated with influential network positions that can later facilitate removals^62^. A useful starting point would be to compare the biological and social traits of invasive species at both native and invasion locations. Linking morphological, physiological, and genetic traits to social behavior can also be useful for examining fisheries catch and for establishing specific regulations to preserve critical social nodes (e.g., individuals with traits that provide more mating opportunities) where fishing practices permit.

### Network comparisons

The third promising research opportunity is the comparison of social networks before and after, or during, natural or anthropogenic perturbation. For instance, the impact of scuba-diving or provisioning activities can be assessed by monitoring the temporal social networks for focal species during periods with and without tourism^48^. Similarly, tracking social interactions before and after the implementation of MPAs, or in response to an illegal fishing event, will help to determine how resilient or responsive populations are to fishing and/or protection. The same approach could be used to monitor changes after natural impacts such as cyclones or heatwaves. To understand the specific impact of fisheries future studies could also include experimental removals on selected individuals in the wild simulating fishing to assess the stability and resilience of social network structure. Although this may represent a logistical challenge, removals could be performed in sedentary and easy-to-catch species such as clown fish, wrasses or cichlids^63^. In addition, some studies have started to show the potential of SNA to understand fishing effects on target populations through simulations. For example, Mourier et al.^64^ showed how a network of black-tip reef sharks, *Carcharhinus melanopterus*, was generally resilient to the removal of nodes with high centrality (i.e., individuals with more interactions). Comparing social structure before and after an impact might also indicate how social structures rewire after human disturbances^65^ (Fig. 4). In combination with movement analyses (e.g., spatial networks), this information should reveal the capacity of different conservation measures to restore the social connectivity of populations. Comparing social networks of local communities before and after reintroduction can also inform the success of such initiatives by detecting alteration in social structure or tracking the social integration of the reintroduced individuals.

### Integrated laboratory and field approaches

Importantly, recognition of the research conducted under captive conditions (e.g., in teasing apart some of the mechanistic drivers of sociality in fishes) and how laboratory and field-based studies might best be integrated, will help to facilitate progress in monitoring the social networks of commercial or endangered species. Some commercially important species already have a wild and captive component to their management, making them highly amenable to integrated research. As an example, species commonly reared in aquaculture such as freshwater eels (*Anguilla spp*.) that are of significant commercial importance and highly threatened globally^66^, may present an opportunity to establish research that considers social behavior in both captive and wild conditions^67^. Behavioral assays, conducted in the laboratory, can also help to determine the factors that dictate aggregation in these complex fishes. Experimental glass (juvenile) and yellow (subadult) eels, housed in captivity, might then be released with long-term conventional or electronic tags to understand how individual social network metrics impact survival and downstream migration, as well as growth rates from an aquaculture management perspective.

## Conclusions

Many population-level processes of fish are influenced by social interactions between individuals, yet social behavior has traditionally not been considered in the conservation and management of fish populations. Here we discuss evidence, suggest research priorities, and provide examples of how SNA may inform resource management and conservation actions in areas such as invasion biology, sustainable fishing, MPAs, and restocking. Many of these ideas are burgeoning and a clear road map to achieving these goals is not yet laid out. Recent technological developments, as well as useful examples from terrestrial species conservation, however, provide a wealth of exciting opportunities to incorporate sociality into applied fish ecology, including the tracking of free-ranging animals and controlled laboratory experiments. It is our intention here to stimulate renewed interest and empirical work on the social structure of fish, as we attempt to safeguard fish stocks under the increasing impacts of climate change.

## Supplementary information

Supplementary material
