# Supplementary material for: Social networks and the conservation of fish
Source: Commun Biol. 2022 Feb 28;5:178. doi: 10.1038/s42003-022-03138-w (PMC8885690; doi:10.1038/s42003-022-03138-w)
Supplement: Supplementary file 1 — Supplementary material [file 42003_2022_3138_MOESM1_ESM.pdf]

# Supplementary material

**Supplementary Table 1.** Studies that have investigated social network structure of fish in the wild.

| Reference | Common name | Scientific name | Topic | Environment | Duration | Method to infer associations | Type of species |
|---|---|---|---|---|---|---|---|
| Croft *et al.* 2004 | Guppy | *Poecilia reticulata* | Social network description | Freshwater | 7 days | Mark-recapture | Model |
| Croft *et al.* 2005 | Guppy and three-spined stickleback | *Poecilia reticulata, Gasterosteus aculeatus* | Assortative interactions and social networks | Freshwater | 7 days | Mark-recapture | Model |
| Croft *et al.* 2006 | Guppy | *Poecilia reticulata* | Cooperative interactions | Freshwater | 31 days | Mark-recapture | Model |
| Croft *et al.* 2009 | Guppy | *Poecilia reticulata* | Social networks and behavioral traits | Freshwater | 9 days | Mark-recapture | Model |
| Krause *et al.* 2009 | Eagle ray | *Aetobatus narinari* | MCMC test of null models | Marine | 20 days | Observations | Near threatened |
| Beyer *et al.* 2010 | Sunbleak | *Leucaspius delineatus* | Invasion success | Freshwater | Several weeks | Lethal sampling | Invasive |
| Guttridge *et al.* 2011 | Lemon shark | *Negaprion brevirostris* | Assortative interactions and leadership | Marine | 2 years | Observations | Near threatened |
| Croft *et al.* 2012 | Guppy | *Poecilia reticulata* | Relatedness and social networks | Freshwater | 12 days | Mark-recapture | Model |
| Mourier *et al.* 2012 | Black tip reef shark | *Carcharhinus melanopterus* | Evidence of social communities | Marine | 2 years | Observations | Vulnerable |
| Stehfest *et al.* 2013 | Yellow fin tuna | *Thunnus albacares* | Association network in FADs | Marine | 3 years | Telemetry | Near threatened |
| Wilson *et al.* 2014 | Guppy | *Poecilia reticulata* | Dynamics social networks | Freshwater | 15 days | Observations | Model |
| Wilson *et al.* 2015b | Guppy | *Poecilia reticulata* | Social networks in changing environments | Freshwater | 2-3 observation days | Observations | Model |
| Wilson *et al.* 2015a | Lemon shark | *Negaprion brevirostris* | Group living and personality | Marine | 8 days | Observations | Near threatened |
| Armansin *et al.* 2016 | Spotted woggegong shark | *Orectolobus maculatus* | Characterization of social patterns. | Marine | 15 months | Telemetry | Least concern |
| Findlay *et al.* 2016 | White shark | *Carcharodon carcharias* | Assessing social interactions vs spatial proximity | Marine | 6 years | Observations | Vulnerable |
| Haulsee *et al.* | Sand tiger shark | *Carcharias taurus* | Fission-fusion patterns and | Marine | 1 year | Telemetry | Vulnerable |

| | | | | | | | |
|---|---|---|---|---|---|---|---|
| 2016 | | | conservation | | | | |
| Jacoby *et al.* 2016 | Grey reef shark | *Carcharhinus amblyrhynchos* | Inferring leadership | Marine | 6 months | Telemetry | Endangered |
| Krause *et al.* 2017 | Guppy | *Poecilia reticulata* | Consistency of social positions | Freshwater | 2 days | Observations | Model |
| Cardoso *et al.* 2017 | Peacock blenny | *Salaria pavo* | Fertilization success | Marine | 2 months | Observations | Least concern |
| McNaughton 2017 | Lake sturgeon | *Acipenser fulvescens* | Interaction between groups | Freshwater | 5 years | Telemetry | Commercial interest |
| Mourier *et al.* 2017b | Black tip reef shark | *Carcharhinus melanopterus* | Robustness to fishing | Marine | 2.5 years | Observations and Mark-recapture | Vulnerable |
| Mourier *et al.* 2017a | Port-Jackson shark | *Heterodontus portusjacksoni* | Methodology in recording interactions and social preferences | Marine | 2 years | Telemetry | Least concern |
| Brena *et al.* 2018 | Sicklefin lemon shark | *Negaprion acutidens* | Social tolerance and dominance hierarchy | Marine | 52 days | Observations | Vulnerable |
| Mourier *et al.* 2019 | Grey and black tip reef sharks | *Carcharhinus amblyrhynchos, Carcharhinus melanopterus* | Multilayer perspective spatial & social networks | Marine | 10-13 months | Telemetry | Endangered, Vulnerable |
| Perryman *et al.* 2019 | Reef manta ray | *Mobula alfredi* | Social preferences and network structure | Marine | 5 years | Observations | Vulnerable |
| Schilds *et al.* 2019 | White shark | *Carcharodon carcharias* | Non-random co-occurrences | Marine | 4.5 years | Observations | Vulnerable |
| Franks *et al.* 2020 | Reef manta ray | *Mobula alfredi* | Difference in gregariousness between the sexes | Marine | 5 years | Telemetry | Vulnerable |
| Lilly *et al.* 2020 | Atlantic sturgeon | *Acipenser oxyrinchus oxyrinchus* | Preferential co-occurrences and management | Marine | 2 years | Telemetry | Near threatened |
| Papastamatiou *et al.* 2020 | Grey reef shark | *Carcharhinus amblyrhynchos* | Long-term social stability | Marine | 4 years | Telemetry | Endangered |
| Mourier and Planes 2021 | Blacktip reef shark | *Carcharhinus melanopterus* | Relatedness and social networks | Marine | 2 years | Observations | Vulnerable |
| Jacoby *et al.* 2021 | Tiger shark | *Galeocerdo cuvier* | Impact of provisioning | Marine | 3 years | Telemetry | Vulnerable |

**Supplementary References**


Armansin NC, Lee KA, Huveneers C, and Harcourt RG. 2016. Integrating social network analysis and fine-scale positioning to characterize the associations of a benthic shark. *Anim Behav* **115**: 245–58.

Beyer K, Gozlan RE, and Copp GH. 2010. Social network properties within a fish assemblage invaded by non-native sunbleak *Leucaspius delineatus*. *Ecol Modell* **221**: 2118–22.

Brena PF, Mourier J, Planes S, and Clua EE. 2018. Concede or clash? Solitary sharks competing for food assess rivals to decide. *Proc R Soc B Biol Sci* **285**.

Cardoso SD, Faustino AI, Costa SS, *et al.* 2017. Social network predicts loss of fertilizations in nesting males of a fish with alternative reproductive tactics. *Acta Ethol* **20**: 59–68.

Croft DP, Hamilton PB, Darden SK, *et al.* 2012. The role of relatedness in structuring the social network of a wild guppy population. *Oecologia* **170**: 955–63.

Croft DP, James R, Thomas POR, *et al.* 2006. Social structure and co-operative interactions in a wild population of guppies (*Poecilia reticulata*). *Behav Ecol Sociobiol* **59**: 644–50.

Croft DP, James R, Ward AJW, *et al.* 2005. Assortative interactions and social networks in fish. *Oecologia* **143**: 211–9.

Croft DP, Krause J, Darden SK, *et al.* 2009. Behavioural trait assortment in a social network: patterns and implications. *Behav Ecol Sociobiol* **63**: 1495–503.

Croft DP, Krause J, and James R. 2004. Social networks in the guppy (*Poecilia reticulata*). *Proc R Soc London Ser B Biol Sci* **271**: S516–9.

Findlay R, Gennari E, Cantor M, and Tittensor DP. 2016. How solitary are white sharks: social interactions or just spatial proximity? *Behav Ecol Sociobiol* **70**: 1735–44.

Franks DW, Weiss MN, Silk MJ, *et al.* 2020. Calculating effect sizes in animal social network analysis. *Methods Ecol Evol* **12**: 33–41.

Guttridge TL, Gruber SH, DiBattista JD, *et al.* 2011. Assortative interactions and leadership in a free-ranging population of juvenile lemon shark *Negaprion brevirostris*. *Mar Ecol Prog Ser* **423**: 235–45.

Haulsee DE, Fox DA, Breece MW, *et al.* 2016. Social Network Analysis Reveals Potential Fission-Fusion Behavior in a Shark. *Sci Rep* **6**.

Jacoby DMP, Papastamatiou YP, and Freeman R. 2016. Inferring animal social networks and leadership: applications for passive monitoring arrays. *J R Soc Interface* **13**: 20160676.

Jacoby, DMP, Fairbairn, BS, Frazier, BS, Gallagher, AJ, Heithaus, MR, Cooke, SJ, & Hammerschlag, N (2021). Social Network Analysis Reveals the Subtle Impacts of Tourist Provisioning on the Social Behavior of a

Generalist Marine Apex Predator. Frontiers in Marine Science, 1202.

Krause S, Mattner L, James R, *et al.* 2009. Social network analysis and valid Markov chain Monte Carlo tests of null models. *Behav Ecol Sociobiol* **63**: 1089–96.

Krause S, Wilson ADM, Ramnarine IW, *et al.* 2017. Guppies occupy consistent positions in social networks: Mechanisms and consequences. *Behav Ecol* **28**: 429–38.

Lilly J, McLean MF, Dadswell MJ, *et al.* 2020. Use of social network analysis to examine preferential co-occurrences in Atlantic Sturgeon *Acipenser oxyrinchus oxyrinchus* Mitchill, 1815. *Anim Biotelemetry* **8**: 14.

McNaughton B. 2017. Social network analysis of lake sturgeon in the Namakan River and Reservoir, Fort Frances District, Ontario.

Mourier J, Bass NC, Guttridge TL, *et al.* 2017a. Does detection range matter for inferring social networks in a benthic shark using acoustic telemetry? *R Soc open Sci* **4**: 170485.

Mourier J, Brown C, and Planes S. 2017b. Learning and robustness to catch-and-release fishing in a shark social network. *Biol Lett* **13**: 20160824.

Mourier J, Ledee EJI, and Jacoby DMP. 2019. A multilayer perspective for inferring spatial and social functioning in animal movement networks. *bioRxiv*: 749085.

Mourier J and Planes S. 2021. Kinship does not predict the structure of a shark social network. *Behav Ecol* **in press**.

Mourier J, Vercelloni J, and Planes S. 2012. Evidence of social communities in a spatially structured network of a free-ranging shark species. *Anim Behav* **83**: 389–401.

Papastamatiou YP, Bodey TW, Caselle JE, *et al.* 2020. Multiyear social stability and social information use in reef sharks with diel fission-fusion dynamics: Shark sociality and information use. *Proc R Soc B Biol Sci* **287**.

Perryman RJY, Venables SK, Tapilatu RF, *et al.* 2019. Social preferences and network structure in a population of reef manta rays. *Behav Ecol Sociobiol* **73**: 114.

Schilds A, Mourier J, Huveneers C, *et al.* 2019. Evidence for non-random co-occurrences in a white shark aggregation. *Behav Ecol Sociobiol* **73**: 1–12.

Stehfest KM, Patterson TA, Dagorn L, *et al.* 2013. Network analysis of acoustic tracking data reveals the structure and stability of fish aggregations in the ocean. *Anim Behav* **85**: 839–48.

Wilson ADM, Brownscombe JW, Krause J, *et al.* 2015a. Integrating network analysis, sensor tags, and observation to understand shark ecology and behavior. *Behav Ecol* **26**: 1577–86.

Wilson ADM, Krause S, James R, *et al.* 2014. Dynamic social networks in guppies (*Poecilia reticulata*). *Behav Ecol Sociobiol* **68**: 915–25.

Wilson ADM, Krause S, Ramnarine IW, *et al.* 2015b. Social networks in changing environments. *Behav Ecol Sociobiol* **69**: 1617–29.
